# Novel and *De Novo* Mutations Extend Association of *POU3F4* with Distinct Clinical and Radiological Phenotype of Hearing Loss

**DOI:** 10.1371/journal.pone.0166618

**Published:** 2016-12-12

**Authors:** Agnieszka Pollak, Urszula Lechowicz, Anna Kędra, Piotr Stawiński, Małgorzata Rydzanicz, Mariusz Furmanek, Małgorzata Brzozowska, Maciej Mrówka, Henryk Skarżyński, Piotr H. Skarżyński, Monika Ołdak, Rafał Płoski

**Affiliations:** 1 Department of Genetics, Institute of Physiology and Pathology of Hearing, Warsaw, Poland; 2 Department of Medical Genetics, Warsaw Medical University, Warsaw, Poland; 3 Postgraduate School of Molecular Medicine, Warsaw Medical University, Warsaw, Poland; 4 Bioimaging Research Center, World Hearing Centre, Institute of Physiology and Pathology of Hearing, Warsaw/Kajetany, Poland; 5 Department of Radiology and Diagnostic Imaging, Medical Centre for Postgraduate Education, Warsaw, Poland; 6 Department of Forensic Medicine, Warsaw Medical University, Warsaw, Poland; 7 Oto-Rhino-Laryngology Surgery Clinic, Institute of Physiology and Pathology of Hearing, Warsaw/Kajetany, Poland; 8 Department of Heart Failure and Cardiac Rehabilitation, Medical University of Warsaw, Warsaw, Poland; 9 Institute of Sensory Organs, Kajetany, Poland; Sant Joan de Déu Children's Hospital, SPAIN

## Abstract

*POU3F4* mutations (DFNX2) are the most prevalent among non-syndromic X-linked hearing loss (HL) identified to date. Clinical manifestations of DFNX2 usually comprise congenital HL either sensorineural or mixed, a tendency towards perilymphatic gusher during otologic surgery and temporal bone malformations. The aim of the present study was to screen for *POU3F4* mutations in a group of 30 subjects with a suggestive clinical phenotype as well as a group (N = 1671–2018) of unselected hearing loss patients. We also planned to analyze audiological and radiological features in patients with HL caused by *POU3F4* defects. The molecular techniques used to detect *POU3F4* mutations included whole exome sequencing (WES), Sanger sequencing and real-time polymerase chain reaction. Hearing status was assessed with pure-tone audiometry and auditory brainstem response. Computer tomography scans were evaluated to define the pattern of structural changes in the temporal bones. Six novel (p.Gln27*, p.Glu187*, p.Leu217*, p.Gln275*, p.Gln306*, p.Val324Asp) and two known (p.Ala116fs141*, p.Leu208*) *POU3F4* mutations were detected in the studied cohort. All probands with *POU3F4* defects suffered from bilateral, prelingual, severe to profound HL. Morphological changes of the temporal bone in these patients presented a similar pattern, including malformations of the internal auditory canal, vestibular aqueduct, modiolus and vestibule. Despite different localization in the *POU3F4* gene all mutations severely impair the protein structure affecting at least one functional POU3F4 domain, and results in similar and severe clinical manifestations. Sequencing of the entire *POU3F4* gene is recommended in patients with characteristic temporal bone malformations. Results of *POU3F4* mutation testing are important not only for a proper genetic counseling, but also for adequate preparation and conduction of a surgical procedure.

## Introduction

Hearing loss (HL) is the most common sensory deficit in humans [1]. Approximately 50–60% of all congenital HL cases are associated with genetic factors [2–4]. The majority of them have autosomal recessive inheritance, however, other types of inheritance also occur, including the X-linked type related to five loci (DFNX1-4 and DFNX6) and four genes (*PRPS1*, *POU3F4*, *SMPX*, *COL4A6*) [5–9]. Additionally, Mohr-Tranebjaerg syndrome (DFN1), caused by mutations in DDP gene is also linked to X chromosome [10]. The most significant of the DFNX genes is *POU3F4* (DFNX2) (MIM*304400) [5] with 63 causative mutations reported so far (Human Gene Mutation Database, accessed April 2016). *POU3F4* encodes a transcription factor playing a crucial role in the neuronal differentiation of mesencephalic neural stem cells and the maturation of neurons in newborns [11]. *POU3F4* is a member of family of POU domain transcription factors with two recognized domains: POU-specific domain and POU-homeodomain, both of which have helix-turn helix pattern and determine DNA specificity and binding [12]. To date according to The Human Gene Mutation Database (HGMD) 44 point mutations have been described in *POU3F4*, among them 30 missense, 11 small deletions and 3 small insertions. Furthermore, deletions of the whole gene as well as deletions, inversions and duplications upstream of the gene (containing the putative regulatory elements) were reported [13]. *POU3F4* is widely expressed in the developing neural tube and subsequently restricted to a few regions of the adult forebrain, supraoptic and paraventricular nuclei of the hypothalamus [12].

Patients with *POU3F4* mutations present with severe to profound sensorineural HL, occasionally with a conductive component, and a variable age of onset. Bilateral malformations of the vestibule, enlarged internal auditory canal and vestibular aqueduct, and underdeveloped cochlear modiolus, jointly defined as incomplete partition type III (IP3), as well as a tendency towards perilymphatic gusher and very rare oozing during inner ear surgery are reported in these patients [14–16].

The aim of our study was to establish prevalence of *POU3F4* mutations and assess their clinical manifestation among selected and unselected HI patients. While testing of a large cohort of two thousand males with different HL levels we identified *POU3F4* mutations in patients with a particular phenotype corresponding to IP3 malformation. Surprisingly, most of the detected mutations were novel adding to the emerging heterogeneity of the DFNX2 locus.

## Materials and Methods

The study was approved by the Ethics Committee at the Institute of Physiology and Pathology of Hearing (IFPS). All procedures were followed in accordance with the ethical standards of the responsible committee on human experimentation and with the Helsinki Declaration of 1975, as revised in 2000 (5). Informed, written consent was obtained from all patients being included in the study.

### Patients

At the first stage two male probands (families B and C) with prelingual HL and a possible X-linked type of inheritance were studied by whole exome sequencing (WES). After finding and confirming by Sanger sequencing the *POU3F4* mutations in both probands we selected additional 28 unrelated patients (23 males and 5 females) in whom direct Sanger sequencing of the whole *POU3F4* coding region was performed. These subjects were retrieved from the clinical database of the IFPS using the keywords: abundant fluid leak, liquorrhoea, gusher, perilymphatic leak, IP3 malformation. Both patients (family B and C) selected for WES also fulfilled the criteria used for database searching. In all probands (n = 30) we have performed sequencing of the whole coding region of the *GJB2* gene, as well as testing for *GJB6* deletions and two most common mitochondrial mutations (m. A1555G, m.A3243G) according to the best practice guidelines for the molecular genetics diagnosis of HL [17].

**Fig 1:**
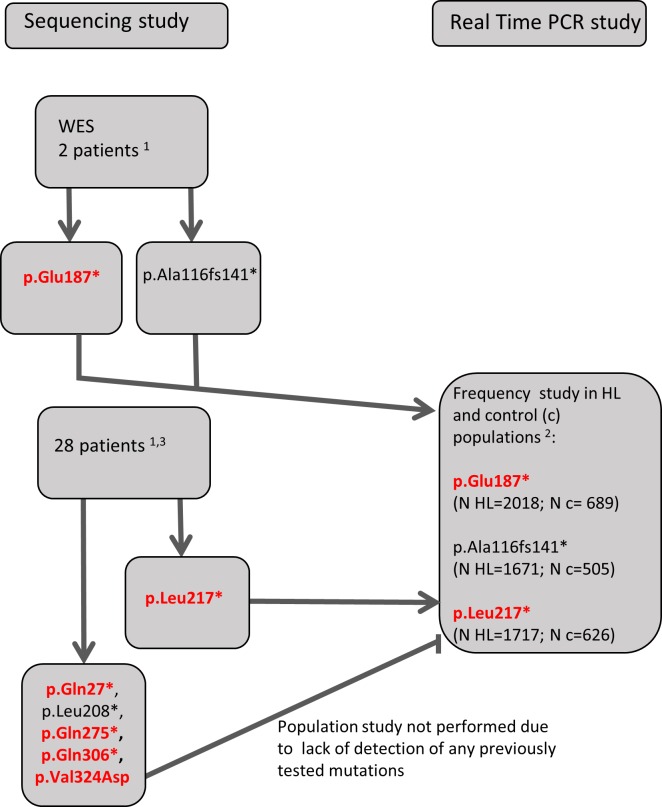
Scheme of the screening strategy. Novel mutations are bolded and marked in red. ^1^ tested previously for all *GJB2* mutations, *GJB6* deletions (del(GJB6-D13S1830) and del(GJB6-D13S1853)), m.A1555G and m.A3243G ^2^HL patients previously tested for 6 *GJB2* mutations (c.-23+1G>A, c.35delG, c.167delT, c.313_326del, c.334_335delAA, c.358_360delGAG), *GJB6* del(GJB6-D13S1830) and m.1555A>G, m.3243A>G) ^3^selected from clinical database with the following keywords: abundant fluid leak, liquorrhoea, gusher, perilymphatic leak, IP3 malformation

In parallel, we performed real-time PCR screening for three of the *POU3F4* mutations among a group derived from 10,000 patients diagnosed at the Department of Genetics IFPS. We selected 2 mutations which were found during WES as well as the third mutation (p.Leu217*) which was the first one found during screening by Sanger sequencing. The subjects tested by real-time PCR were all males with different age of onset and severity of bilateral HL with common reasons for HL excluded (as described above). They had all been previously tested for 6 *GJB2* mutations most common in Polish population i.e. *GJB2* (c.-23+1G>A, c.35delG, c.167delT, c.313_326del, c.334_335delAA, c.358_360delGAG), *GJB6* del(GJB6-D13S1830) and mitochondrial DNA (m.1555A>G, m.3243A>G) which together account for more than 80% of genetically related HL in our population. Also, major environmental risk factors (i.e. severe prematurity, congenital rubella, mumps or cytomegalovirus infection, severe neonatal hyperbilirubinaemia or ototoxic drugs) were excluded in all these patients. Individual *POU3F4* mutations (in parentheses) were tested among 2018 (NM_000307.4:c.559G>T, p.Glu187*), 1717 (NM_000307.4:c.650T>A, p.Leu217*)) and 1671 (NM_000307.4:c.346delG, p.Ala116fs141*) subjects, respectively. Detailed scheme of the mutation detection and screening strategy is shown in Fig 1.

### Control group

Control DNA samples came from a set of anonymized samples from the Medical University of Warsaw, Department of Forensic Medicine collected from unrelated adult individuals born in Central Poland who underwent paternity testing (female/male ratio 1:1). Hearing status of these individuals was unknown. The number of subjects tested for each *POU3F4* mutation was as follows: 689 (p.Glu187*), 626 (p.Leu217*) and 505 (p.Ala116fs141*).

### DNA isolation and Sanger sequencing

DNA was isolated from peripheral blood by standard salting out method [18] or from buccal swabs with Maxwell 16 instrument (Promega, Madison, USA). PCR primers were designed with Primer 3 software [19, 20] based on the NM_000307.4 reference sequence. PCR primers sequences and thermal condition of the reactions are available on request.

### Whole Exome Sequencing (WES)

WES was performed on Illumina HiSeq 1500 platform (Illumina Inc., San Diego, CA, USA). Library was prepared with TruSeq Exome Enrichment kit (Illumina Inc.) according to the manufacturer’s protocol. The samples were run on 1/4 of a lane on HiSeq 1500 using 2x100 bp paired-end reads and sequenced so that 90% of target was covered 20x or more. The data were analyzed as described previously [21], Briefly, raw HiSeq reads were transformed to FASTQ files using bcl2fastq software. After the adapter trimming and removal of low quality data, reads were aligned to the human genome using BWA software. After duplicate marking, local realignment and reads recalibration step, the variant calling was performed using the Haplotype Caller from GATK framework, followed by variant annotation using several databases, including EXaC, dbSNP, dbNSFP, 1000genomes and in-house variant frequencies database. For further analysis only variants with low population frequency (<1%) were considered. In the first stage of the analysis we looked at genes reported to date as associated with HL. In the subsequent step we performed wide, unfocused, analysis of rare, homozygous or compound heterozygous variants. The data from next-generation sequencing (WES) in the context of Polish law can be used for personal identification. Due to this we do not have permission to deposit such data in public databases, but data can be made available upon request to the corresponding authors. Population frequencies of *POU3F4* variants were obtained from the database of the Exome Aggregation Consortium (ExAC) (http://exac.broadinstitute.org) (accessed 04/2016) and the in-house database of WES data from the Polish population (n = 816). For pathogenicity prediction of the p.Val324Asp mutation the following algorithms were used: SIFT MutationTaster2, PolyPhen2 and Provean.

### Real time PCR screening

All real-time allelic discrimination screenings were performed with assay on demands (Life Technologies, Carlsbad, CA, USA) on the Viia7 (Life Technologies) apparatus.

### Audiological and imaging studies

Hearing levels were determined by pure-tone audiometry at frequencies of 500 Hz, 1 kHz, 2 kHz, 4 kHz, and 8 kHz or by extended frequency specified Auditory Brainstem Response (ABR) tests. Distinction between severe and profound HL was based on the American National Standards Institute (ANSI) and International Standards Organization (ISO) scale introduced in 1965 (71–90 dB HL was defined as severe, whereas more than 90 dB HL was regarded as profound). Temporal bone computer tomography (CT) examination was performed using Siemens CT Definitions AS system (Siemens, Munich, Germany).

## Results

### Identification of *POU3F4* mutations

**Fig 2:**
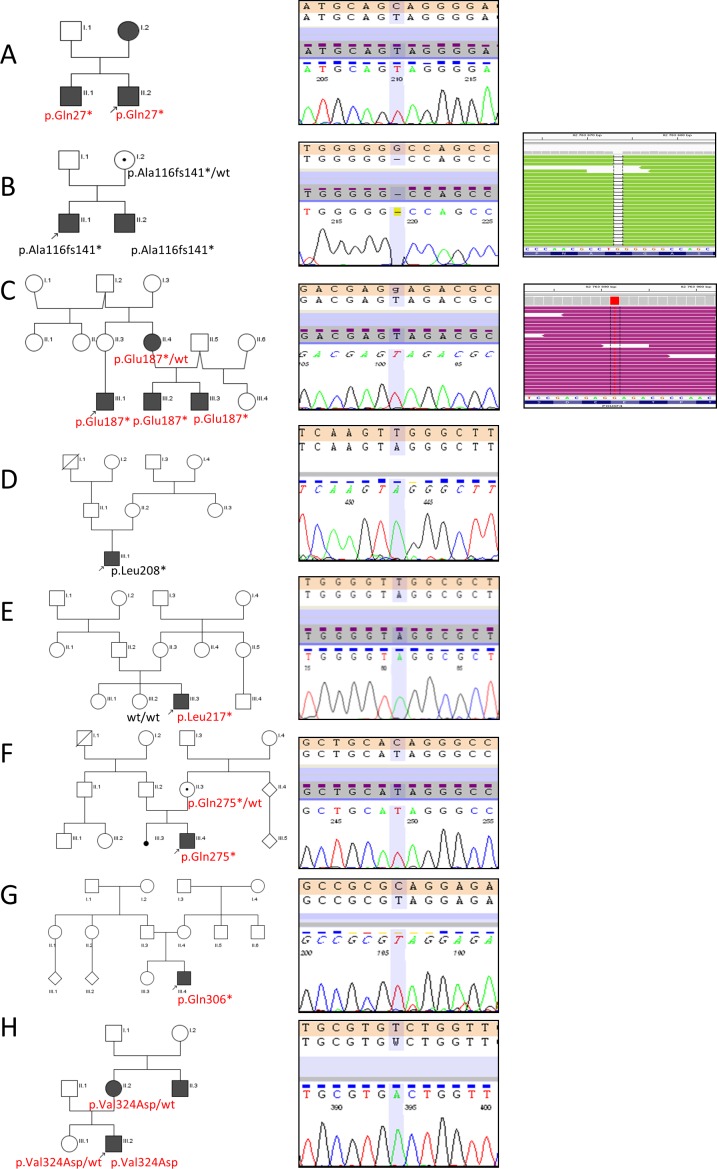
Identification of *POU3F4* mutations. Family pedigrees (left panel) accompanied by chromatograms from *POU3F4* Sanger sequencing (middle panel) and Integrative Genomics Viewer (IGV) views (right panel) are shown. Circles represent females and squares represent males. The filled symbols indicate affected individuals and dots inside symbols indicate female carriers. Probands are marked with arrows. *POU3F4* mutations (novel in red, known in black) identified in the respective families (A-H) are given below the symbols.

WES analysis in two probands revealed two different *POU3F4* mutations. One of them (p.Glu187*/c.559G>T) was novel and the second one (p.Ala116fs141*/c.346delG) was previously reported. Both detected mutations were verified by direct Sanger sequencing. Sanger Sequencing was also used in family studies. Additionally, six other mutations i.e.: p.Gln27*/c.79C>T, p.Leu208*/c.623T>A, p.Leu217*/c.650T>A, p.Gln275*/c.823C>T; p.Gln306*/c.916C>T and p.Val324Asp/c.971T>A were found in patients selected for direct Sanger sequencing of the entire coding sequence of *POU3F4* gene (6/28; 21.4%). In the whole group (n = 30) a total of eight different *POU3F4* mutations were detected (Fig 2). Apart from p.Ala116fs141* and p.Leu208* all detected *POU3F4* variants have not been reported to date. None of the eight identified *POU3F4* variants were found in the Exome Aggregation Consortium (ExAC, Cambridge, MA, USA) (http://exac.broadinstitute.org; accessed 04/2016) (n = 60706), nor in our exome database of the Polish population (n = 816). Moreover, none of the three tested *POU3F4* mutations, p.Glu187*, p.Leu217*, p.Ala116fs141*, were found in the control group of Polish origin (n~500).

To avoid a selective approach for studying *POU3F4* mutation exclusively in patients with a particular phenotype, we screened a large, unselected group of HL male patients (n~1700) for the presence of p.Glu187*, p.Leu217*, p.Ala116fs141*. None of the studied mutations was found in any of the screened patients.

All presented mutations, apart from p.Val324Asp, lead to the premature termination of translation. The p.Val324Asp mutation affects the second putative nuclear localization signal (NLS) of the POU homeodomain, and based on prediction algorithms is considered damaging and disease causing (Table 1).

**Table 1:** Results of pathogenicity algorithms for the p.Val324Asp mutation.

Algorithm	SIFT	MutationTaster2	PolyPhen2	Provean
Score	damaging	disease causing	Probably damaging (score 1,000*)	Deleterious (score -6,852**)

*(sensitivity: 0.00; specificity: 1.00)

**(cutoff = -2.5)

**Fig 3:**
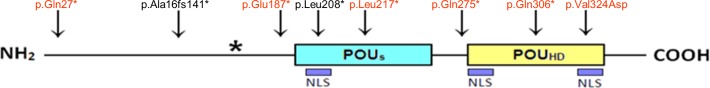
Schematic representation of the POU3F4 protein. POU specific domain (POU_S_), POU homeodomain (POU_HD_) and putative nuclear localization signals (NLS) are depicted. Arrows indicate the positions of all mutations identified in this study (novel in red, known in black) in respect to the POU3F4 protein. The asterisk marks the location of a premature termination in p.Ala116fs141* mutation.

Using Sanger sequencing, we confirmed a carrier status of p.Ala116fs141* (family B), p.Gln275* (family F), and p.Val324Asp (family H) mutations in mothers of the respective probands. Surprisingly, the p.Leu217* (family E) mutation was not found in the proband’s mother and was classified as a *de novo* event. For the remaining four probands DNA samples from their parents were not available for testing. Localization of all detected mutations in the POU3F4 protein is shown in Fig 3.

### Clinical characterization of patients carrying *POU3F4* mutations

Based only on pedigrees, among all analyzed families there were no obvious cases of X-linked HL. In the majority of pedigrees the mode of HL inheritance could be considered as autosomal recessive or dominant (Fig 2 and Table 2). All affected males regardless of the *POU3F4* mutation type suffered from bilateral, prelingual HL (age of onset varied from congenital to 2 y.). The level of HL ranged from moderate to profound. Two cases (Table 2, family B and H) presented a mixed type (sensorineural and conductive) of HL, in the remaining patients only sensorineural deafness was detected. During surgeries (cochlear implantation (CI) for probands from families A, D, E, F and explorative tympanotomy for probands from families B and H) perilymphatic gusher occurred.

**Table 2:** Clinical characterization of patients harboring *POU3F4* mutations.

	p.Gln27* family A	p.Ala116fs141* family B	p.Glu187* family C	p.Leu208* family D	p.Leu217* family E	p.Gln275* family F	p.Gln306* family G	p.Val324Asp family H
Pedigree type	AD	AR or X-linked	AD	sporadic case unspecific	sporadic case unspecific	sporadic case unspecific	sporadic case unspecific	AD
Age of onset	congenital	congenital	1.5 y.o.	congenital	2 y.o.**	congenital	congenital	congenital
Classification	prelingual	prelingual	prelingual	prelingual	prelingual	prelingual	prelingual	prelingual
Level of hearing loss	profound	profound	severe	profound	profound	severe	severe	severe
Type of hearing loss	sensorineural	mixed	sensorineural	sensorineural	sensorineural	sensorineural	sensorineural	mixed
Gusher during surgery	+	+	nd	+	+	+	nd	+
Dilatation of internal auditory canal	+	+	+	+	+	+	+	+
Absent/severely underdeveloped cochlear modiolus	+	+	+	+	+	+	+	+
Distorted vestibule	+	+	+	+	+	+	+	+
Dilatation of vestibular aqueduct	+/-	+	+	-	+	+	+/-	+/-
Third window effect***	+	+	+	+	+	+	+	+
Cochlear implant	+	-	-	+	+	+	-	-

** patient passed newborn hearing screen

***abnormal connection between the subarachnoidal space of the internal auditory canal, nd- no data

**Fig 4:**
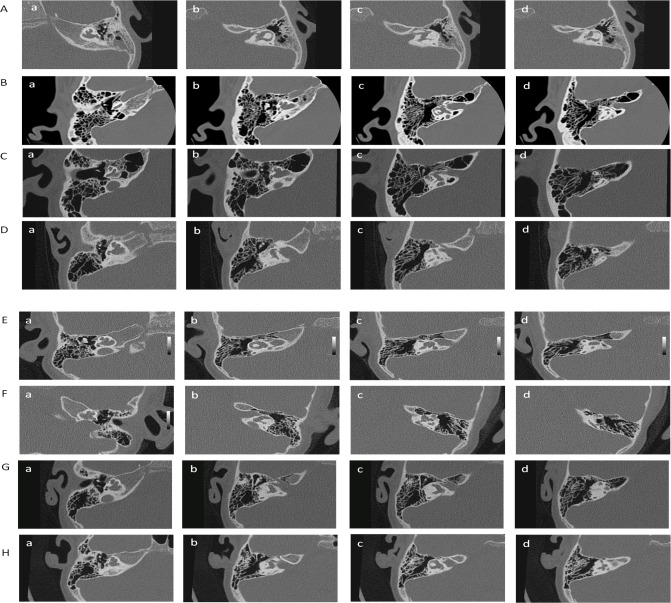
CT of temporal bones in the probands from families with POU3F4 mutations: A- p.Gln27*; B—p.Ala116fs141*; C-p.Glu187*; D- p.Leu208*; E- p.Leu217*; F- p.Gln275*; G- p.Gln306*; H- p.Val324Asp. CT of temporal bone displays widened internal auditory canal (b, c) and inner ear malformations with leading feature of underdeveloped cochlear modiolus with complete absence of its base (a, b). Other features are: distorted vestibule (b, c) with small outpouchings (c, d), widened vestibular aqueduct (c), irregular semicircular ducts (c, d), The interscalar septa are preserved (a, b).

In all probands the temporal bone imaging studies revealed widening of internal auditory canals (IAC) as well as bilateral inner ear malformations. In particular, lack or severely underdeveloped cochlear modiolus, which may result in a fistulous communication between subarachnoid space of the internal auditory canal and perilymphatic space of the inner ear, was noted. Furthermore, distorted vestibule, widened vestibular aqueduct, and dysplastic semicircular ducts were visualized. The observed inner ear malformations are within the range of the IP3 spectrum (Fig 4). The most important phenotypic features of the probands carrying *POU3F4* mutations are summarized in Table 2.

### Clinical characterization of affected female carriers of the *POU3F4* mutations

The proband’s mother from the family A harboring the heterozygous p.Gln27* mutation suffered from bilateral, prelingual, severe, mixed type HL (mean air bone gap 30 dB). The onset of HL at 2 y was associated with the intake of aminoglycosides. Further audiological assessment revealed normal middle ear function, however acoustic reflexes were absent for all tested frequencies in both ears. Due to her pregnancy we could not perform temporal bone CT examination. Although aminoglycoside exposition cannot be excluded as the cause of HL pronounced air-bone gap and bilateral lack of acoustic reflexes may suggest that the inner ear malformations were present in this patient.

The female from family C heterozygous for p.Glu187* mutation complained of HL from the age of 35 y. She did not agree to undergo detailed audiological and temporal bone CT examinations. Based on previous limited audiological results we identified a bilateral mild to moderated HL with a normal middle ear function.

The proband’s mother from family H with heterozygous p.Val324Asp mutation suffered from moderate, postlingual, mixed type HL (mean air bone gap 20 dB) that was diagnosed at 20 y. Temporal bone CT examination revealed bilateral inner ear malformations, i.e. absence of cochlear modiolus, incomplete separation of the cochlear turns, widened vestibule canals and a narrow round window.

## Discussion

We identified six novel and two known *POU3F4* pathogenic mutations in 26.6% (8/30) of all patients selected for either WES or Sanger sequencing. This number increases to 32% (8/25), if we exclude females, taking into account that HL caused by *POU3F4* mutations is inherited in an X-linked pattern with the phenotype in females being significantly milder and present only seldom [22]. All but one of the identified *POU3F4* mutations are predicted to truncate the protein and thus abolish or considerably impair its function. Most of them result in the loss of at least one POU homeodomain, an important part for protein-DNA interactions, with two NLS [23], while the p.Val324Asp mutation affects the NLS site at the carboxy terminus (Fig 3). In the whole analyzed group we didn’t find any deletions involving a part or the whole *POU3F4* gene. Since such deletions should be readily detectable in males, in Polish population point mutations of *POU3F4* gene are apparently more common than large deletions.

All identified *POU3F4* mutations were unique for each family which emphasizes a substantial allelic heterogeneity of *POU3F4*. Our data argues for sequencing of the entire *POU3F4* gene rather than targeted screening for particular mutations. It should also be emphasized that mutations in *POU3F4* may occur *de novo* as well. This was the case for the p.Leu217* and the other previously reported *POU3F4* mutation (p.N244KfsX26) [15]. Thus, direct examination of the carrier status of mothers should be obligatory for a proper genetic counseling.

Our results illustrate that pedigree analysis is often of limited value in detecting *POU3F4*-linked HL. In small families which are common, especially in European countries, it is difficult to distinguish the X-linked inheritance pattern. In four of our families there were only single affected cases. Even in family B with two affected brothers, the pedigree pattern of HL could be interpreted as autosomal recessive, which is the most frequent type of HL inheritance.

The interpretation of pedigree data is further complicated by symptoms occurring in some female carriers. In our study there were three females with HL among five carriers in whom carrier status was molecularly confirmed. The *POU3F4* associated disease was most likely present in one of these subjects as bilateral inner ear malformations were found on CT. In the remaining two subjects such malformations were not tested for (in one the interpretation was further complicated by exposure to aminoglycosides). Our results add to previous reports describing relatively high (up to 40%) prevalence of HL among female carriers of *POU3F4* mutations [22, 24] which may be at odds with current information in OMIM classifying the *POU3F4* disease as sex linked recessive (http://omim.org, accessed 08/2016).

All mutations described in this report, regardless of their status (truncating or missense), are associated with a similar severe phenotype, i.e. bilateral, prelingual severe to profound HL accompanied by bilateral inner ear malformations fulfilling the criteria of IP3 (Fig 4, Table 2). The available data for carrier females suggests diversity in age of onset, which may be due to the type of the mutation (later for the nontruncating mutation) or the presence of the additional environmental factors such as ototoxic drugs in case of truncating mutation. Diversity in the age of onset in carrier females may be also related to biases in X chromosome inactivation although this possibility has been argued against [22].

Patients harboring *POU3F4* mutations are highly predisposed to complications during and after ear surgery due to the absence of the bony and/or soft septum between the cochlea and IAC. It leads to leakage of the cerebrospinal fluid into the middle ear cavity which, in addition to posing problems for surgeon, may cause meningitis [25]. In patients with conductive or mixed HL, who undergo a stapedotomy, the situation gets even more complicated as the development of gusher may lead to a deterioration of hearing or even complete deafness in the ear operated on. Furthermore, cochlear implantation may result in electrode insertion into the IAC without auditory stimulation and risk of facial nerve injury [26]. Imaging studies and *POU3F4* analysis before cochlear implantation can help to avoid intra- and postoperative complications or at least prepare for a designated technique [27–30].

Patients described in this report experienced substantial benefits after CI and one of them is scheduled for a second ear implantation. This may be due to the fact that our implanted patients harbor point mutations which, in contrast to gross deletions resulting in a contiguous gene syndrome, do not cause developmental delay associated with the difficulties in rehabilitation process [31].

HL-associated genes may present a phenotypic diversity as exemplified by the *SLC26A4* mutations which can cause either Pendred Syndrome, or isolated HL with or without enlarged vestibular aqueduct (EVA) [32–34]. Thus, we speculated that *POU3F4* mutations may also lead to a variable phenotype. To verify this hypothesis screening of a large group of males with HL for the p.Glu187*, p.Leu271*, p.Ala116fs141* *POU3F4* mutations was performed. As the mutations were not found in any of the screened patients we conclude that they are either very rare or indeed strongly linked to a particular phenotype. Given the diversity of *POU3F4* mutations sequencing of the whole gene in the whole cohort would have been clearly preferable.

In summary, searching for *POU3F4* mutations is effective among selected patients with particular phenotypic features and it should preferably be based on sequencing of the entire gene, because of the high *POU3F4* allelic heterogeneity. For a proper genetic counseling it is essential to investigate whether the mutation is inherited or *de novo*. Decision about cochlear implantation in *POU3F4* patients should be made with special caution and adequate operation technique should be applied.

## References

[1] Morton CC, Nance WE. Newborn hearing screening—a silent revolution. The New England journal of medicine. 2006;354(20):2151–64. 10.1056/NEJMra050700 16707752

[2] Estivill X, Fortina P, Surrey S, Rabionet R, Melchionda S, D'Agruma L, et al Connexin-26 mutations in sporadic and inherited sensorineural deafness. Lancet. 1998;351(9100):394–8. 10.1016/S0140-6736(97)11124-2 9482292

[3] Pollak A, Skorka A, Mueller-Malesinska M, Kostrzewa G, Kisiel B, Waligora J, et al M34T and V37I mutations in GJB2 associated hearing impairment: evidence for pathogenicity and reduced penetrance. American journal of medical genetics Part A. 2007;143A(21):2534–43. 10.1002/ajmg.a.31982 17935238

[4] Van Camp G, Willems PJ, Smith RJ. Nonsyndromic hearing impairment: unparalleled heterogeneity. American journal of human genetics. 1997;60(4):758–64. PubMed Central PMCID: PMC1712474. 9106521 PMC1712474

[5] Bitner-Glindzicz M, Turnpenny P, Hoglund P, Kaariainen H, Sankila EM, van der Maarel SM, et al Further mutations in Brain 4 (POU3F4) clarify the phenotype in the X-linked deafness, DFN3. Human molecular genetics. 1995;4(8):1467–9. 7581392 10.1093/hmg/4.8.1467

[6] Huebner AK, Gandia M, Frommolt P, Maak A, Wicklein EM, Thiele H, et al Nonsense mutations in SMPX, encoding a protein responsive to physical force, result in X-chromosomal hearing loss. American journal of human genetics. 2011;88(5):621–7. PubMed Central PMCID: PMC3146719. 10.1016/j.ajhg.2011.04.007 21549336 PMC3146719

[7] Liu XZ, Xie D, Yuan HJ, de Brouwer AP, Christodoulou J, Yan D. Hearing loss and PRPS1 mutations: Wide spectrum of phenotypes and potential therapy. International journal of audiology. 2013;52(1):23–8. 10.3109/14992027.2012.736032 23190330 PMC4511087

[8] Rost S, Bach E, Neuner C, Nanda I, Dysek S, Bittner RE, et al Novel form of X-linked nonsyndromic hearing loss with cochlear malformation caused by a mutation in the type IV collagen gene COL4A6. European journal of human genetics: EJHG. 2014;22(2):208–15. PubMed Central PMCID: PMC3895628. 10.1038/ejhg.2013.108 23714752 PMC3895628

[9] Schraders M, Haas SA, Weegerink NJ, Oostrik J, Hu H, Hoefsloot LH, et al Next-generation sequencing identifies mutations of SMPX, which encodes the small muscle protein, X-linked, as a cause of progressive hearing impairment. American journal of human genetics. 2011;88(5):628–34. PubMed Central PMCID: PMC3146715. 10.1016/j.ajhg.2011.04.012 21549342 PMC3146715

[10] Jin H, May M, Tranebjaerg L, Kendall E, Fontan G, Jackson J, et al A novel X-linked gene, DDP, shows mutations in families with deafness (DFN-1), dystonia, mental deficiency and blindness. Nature genetics. 1996;14(2):177–80. 10.1038/ng1096-177 8841189

[11] Tan XF, Qin JB, Jin GH, Tian ML, Li HM, Zhu HX, et al Effects of Brn-4 on the neuronal differentiation of neural stem cells derived from rat midbrain. Cell biology international. 2010;34(9):877–82. 10.1042/CBI20100214 20524937

[12] Mathis JM, Simmons DM, He X, Swanson LW, Rosenfeld MG. Brain 4: a novel mammalian POU domain transcription factor exhibiting restricted brain-specific expression. The EMBO journal. 1992;11(7):2551–61. PubMed Central PMCID: PMC556730. 1628619 10.1002/j.1460-2075.1992.tb05320.x PMC556730

[13] Cremers F, Cremers C, Kremer H. POU3F4 and mixed deafness with temporal bone defect (DFN3). OXFORD MONOGRAPHS ON MEDICAL GENETICS. 2008;54(1):1042–7.

[14] Friedman RA, Bykhovskaya Y, Tu G, Talbot JM, Wilson DF, Parnes LS, et al Molecular analysis of the POU3F4 gene in patients with clinical and radiographic evidence of X-linked mixed deafness with perilymphatic gusher. The Annals of otology, rhinology, and laryngology. 1997;106(4):320–5. 9109724 10.1177/000348949710600411

[15] Moteki H, Shearer AE, Izumi S, Kubota Y, Azaiez H, Booth KT, et al De Novo Mutation in X-Linked Hearing Loss-Associated POU3F4 in a Sporadic Case of Congenital Hearing Loss. The Annals of otology, rhinology, and laryngology. 2015;124 Suppl 1:169S–76S. PubMed Central PMCID: PMC4441833. 10.1177/0003489415575042 PMC4441833 25792666

[16] Sennaroglu L, Sarac S, Ergin T. Surgical results of cochlear implantation in malformed cochlea. Otology & neurotology: official publication of the American Otological Society, American Neurotology Society [and] European Academy of Otology and Neurotology. 2006;27(5):615–23. 10.1097/01.mao.0000224090.94882.b4 16788416

[17] Hoefsloot LH, Roux AF, Bitner-Glindzicz M, contributors to EDbpm. EMQN Best Practice guidelines for diagnostic testing of mutations causing non-syndromic hearing impairment at the DFNB1 locus. European journal of human genetics: EJHG. 2013;21(11):1325–9. PubMed Central PMCID: PMC3798855. 10.1038/ejhg.2013.83 23695287 PMC3798855

[18] Miller SA, Dykes DD, Polesky HF. A simple salting out procedure for extracting DNA from human nucleated cells. Nucleic acids research. 1988;16(3):1215 PubMed Central PMCID: PMC334765. 3344216 10.1093/nar/16.3.1215 PMC334765

[19] Koressaar T, Remm M. Enhancements and modifications of primer design program Primer3. Bioinformatics. 2007;23(10):1289–91. 10.1093/bioinformatics/btm091 17379693

[20] Untergasser A, Cutcutache I, Koressaar T, Ye J, Faircloth BC, Remm M, et al Primer3—new capabilities and interfaces. Nucleic acids research. 2012;40(15):e115 PubMed Central PMCID: PMC3424584. 10.1093/nar/gks596 22730293 PMC3424584

[21] Ploski R, Pollak A, Muller S, Franaszczyk M, Michalak E, Kosinska J, et al Does p.Q247X in TRIM63 cause human hypertrophic cardiomyopathy? Circulation research. 2014;114(2):e2–5. 10.1161/CIRCRESAHA.114.302662 24436435

[22] Marlin S, Moizard MP, David A, Chaissang N, Raynaud M, Jonard L, et al Phenotype and genotype in females with POU3F4 mutations. Clinical genetics. 2009;76(6):558–63. 10.1111/j.1399-0004.2009.01215.x 19930154

[23] Lee HK, Song MH, Kang M, Lee JT, Kong KA, Choi SJ, et al Clinical and molecular characterizations of novel POU3F4 mutations reveal that DFN3 is due to null function of POU3F4 protein. Physiological genomics. 2009;39(3):195–201. 10.1152/physiolgenomics.00100.2009 19671658

[24] Parzefall T, Shivatzki S, Lenz DR, Rathkolb B, Ushakov K, Karfunkel D, et al Cytoplasmic mislocalization of POU3F4 due to novel mutations leads to deafness in humans and mice. Human mutation. 2013;34(8):1102–10. PubMed Central PMCID: PMC3714346. 10.1002/humu.22339 23606368 PMC3714346

[25] Gong WX, Gong RZ, Zhao B. HRCT and MRI findings in X-linked non-syndromic deafness patients with a POU3F4 mutation. International journal of pediatric otorhinolaryngology. 2014;78(10):1756–62. 10.1016/j.ijporl.2014.08.013 25175280

[26] Kumar G, Castillo M, Buchman CA. X-linked stapes gusher: CT findings in one patient. AJNR American journal of neuroradiology. 2003;24(6):1130–2. 12812938 PMC8149040

[27] Skarzynski H. Ten years experience with a new strategy of partial deafness treatment. Journal of Hearing Science. 2012;2(2):11–8.

[28] Skarzynski H, Lorens A, Piotrowska A, Skarzynski PH. Hearing preservation in partial deafness treatment. Medical science monitor: international medical journal of experimental and clinical research. 2010;16(11):CR555–62. 20980961

[29] Skarzynski H, Matusiak M, Lorens A, Furmanek M, Pilka A, Skarzynski PH. Preservation of cochlear structures and hearing when using the Nucleus Slim Straight (CI422) electrode in children. The Journal of laryngology and otology. 2016;130(4):332–9. 10.1017/S0022215115003436 26763105

[30] Stankovic KM, Hennessey AM, Herrmann B, Mankarious LA. Cochlear implantation in children with congenital X-linked deafness due to novel mutations in POU3F4 gene. The Annals of otology, rhinology, and laryngology. 2010;119(12):815–22. 21250553 10.1177/000348941011901205

[31] Song MH, Lee HK, Choi JY, Kim S, Bok J, Kim UK. Clinical evaluation of DFN3 patients with deletions in the POU3F4 locus and detection of carrier female using MLPA. Clinical genetics. 2010;78(6):524–32. 10.1111/j.1399-0004.2010.01426.x 20412083

[32] Duman D, Tekin M. Autosomal recessive nonsyndromic deafness genes: a review. Frontiers in bioscience. 2012;17:2213–36. PubMed Central PMCID: PMC3683827. 10.2741/4046 PMC3683827 22652773

[33] Hilgert N, Smith RJ, Van Camp G. Forty-six genes causing nonsyndromic hearing impairment: which ones should be analyzed in DNA diagnostics? Mutation research. 2009;681(2–3):189–96. PubMed Central PMCID: PMC2847850. 10.1016/j.mrrev.2008.08.002 18804553 PMC2847850

[34] Pourova R, Janousek P, Jurovcik M, Dvorakova M, Malikova M, Raskova D, et al Spectrum and frequency of SLC26A4 mutations among Czech patients with early hearing loss with and without Enlarged Vestibular Aqueduct (EVA). Annals of human genetics. 2010;74(4):299–307. 10.1111/j.1469-1809.2010.00581.x 20597900

